# Evaluating the Use of Dalbavancin for Off-Label Indications

**DOI:** 10.3390/idr14020032

**Published:** 2022-04-11

**Authors:** Katherine Taylor, John Williamson, Vera Luther, Tyler Stone, James Johnson, Zachary Gruss, Courtney Russ-Friedman, Chris Ohl, James Beardsley

**Affiliations:** 1 Wake Forest Baptist Health Department of Pharmacy, Winston-Salem, NC 27157, USA; johnwill@wakehealth.edu (J.W.); tjstone@wakehealth.edu (T.S.); johnsonj@wakehealth.edu (J.J.); jbeardsl@wakehealth.edu (J.B.); 2 Section of Infectious Diseases, Wake Forest School of Medicine, Winston-Salem, NC 27157, USA; vluther@wakehealth.edu (V.L.); crussfri@wakehealth.edu (C.R.-F.); cohl@wakehealth.edu (C.O.); 3 ProMedica Toledo Hospital Department of Pharmacy, Toledo, OH 43606, USA; zachary.gruss@promedica.org

**Keywords:** dalbavancin, osteomyelitis, endocarditis, bacteremia, pharmacoeconomics, cohort studies, historical

## Abstract

(1) Background: Dalbavancin is a long-acting lipoglycopeptide antibiotic approved for skin and soft-tissue infections. Post-marketing experience suggests dalbavancin is being used for off-label indications that normally require long-term intravenous (IV) antibiotics; however, data assessing this off-label usage are limited. The purpose of this study was to evaluate the real-world efficacy, safety, and financial impact of off-label dalbavancin use. (2) Methods: This is a retrospective, observational study conducted within a 4-hospital health system. Adult patients who received dalbavancin from January 2018 to January 2021 for an off-label indication were included. The primary outcome was clinical success at 90 days. Secondary outcomes included safety (nephrotoxicity and hepatotoxicity). A pharmacoeconomic analysis was performed by comparing the cost of dalbavancin to the anticipated cost of patient stay if standard IV therapy was given. (3) Results: Forty-eight patients met study criteria. Indications included osteomyelitis (54%), endocarditis (23%), bacteremia (15%), and prosthetic joint infection (8%). The predominant organism was *S. aureus* (60%), with 42% caused by methicillin-resistant *S. aureus*. Overall, 41 (85%) patients achieved clinical success at 90 days, including 85% with osteomyelitis, 82% with endocarditis, and 86% with bacteremia. There were no instances of nephrotoxicity or hepatotoxicity. Estimated cost avoidance per patient was USD 5313 and USD 1683 if traditional IV therapy would have been completed in the hospital and skilled nursing facility, respectively. (4) Conclusion: Dalbavancin was associated with a relatively high success rate for the treatment of off-label indications and may be a cost-effective alternative to traditional IV antibiotic therapy.

## 1. Introduction

Dalbavancin is a long-acting lipoglycopeptide antibiotic with an FDA-approved indication for acute bacterial skin and skin structure infections (ABSSSIs). While dalbavancin is traditionally used for ABSSSIs, it has also been used off-label to treat patients with serious, Gram-positive infections requiring long-term therapy [1,2,3]. Its 14 day half-life allows patients to receive therapy in the outpatient setting and avoid the need for long-term IV line access. Additionally, dalbavancin has a favorable side effect profile with few adverse effects. The current preferred treatment regimen for complicated Gram-positive infections is long-term IV antibiotic therapy with the possibility to transition to oral therapy to complete treatment for certain indications. Disadvantages of these regimens include the need for long-term IV line access, potential for adverse outcomes related to the IV, therapeutic drug monitoring, as well as an increased risk of medication-related adverse effects. While dalbavancin is not currently the preferred regimen for these infections, it is an attractive alternative antibiotic option for patients who are unable or unlikely to adhere to preferred regimens, have a recent history of IV substance use disorder, have a social situation that is incompatible with home treatment, or are unwilling to receive long-term outpatient IV antibiotics [4,5,6].

Data assessing the off-label use of dalbavancin are limited. There are some studies and case series evaluating its off-label use in osteomyelitis, infective endocarditis, and septic joint infections; however, data evaluating the clinical impact and economic impact of using dalbavancin for these conditions within the United States health system are minimal. One study from the University of Colorado Health suggests that dalbavancin and oritavancin, another long-acting lipoglycopeptide antibiotic, may be appropriate and efficacious therapeutic options for on- and off-label Gram-positive infections [4]. Although the sample size was small (46 evaluable patients), failure rates were low at 15%, and there were significant reductions in hospital length of stay and amount of time spent receiving IV infusions. Additionally, there was an estimated cost savings of USD 17,204 per patient. Given the substantial financial impact dalbavancin could have on health care systems in the United States, it is imperative that we develop a better understanding of the effectiveness of dalbavancin when used in a real-world setting for off-label infections.

Wake Forest Baptist Health (WFBH) is a four-hospital health system that includes an 885 bed academic medical center. At WFBH, dalbavancin is utilized for Gram-positive infections in patients who are not candidates for standard therapy. The process to receive dalbavancin at WFBH is stringent and highly regulated. All dalbavancin use requires approval from infectious disease stewardship clinicians, and candidates for dalbavancin are restricted to those in whom oral therapy and home IV therapy are not suitable choices^8^. The purpose of this study was to evaluate the efficacy and safety of off-label dalbavancin in patients with Gram-positive infections requiring long-term antibiotics. Additionally, the potential cost impact of using dalbavancin compared to traditional therapy was evaluated.

## 2. Materials and Methods

This was a single health system, retrospective, observational study. Patients who received dalbavancin from January 2018 to January 2021 were identified for screening via a report from the electronic health record (EHR). Patients were included if they were ≥18 years of age and received dalbavancin for an indication that was not FDA approved (i.e., not ABSSSI). Patients were excluded if they were pregnant or if they had a concomitant infection caused by a pathogen not expected to be susceptible to dalbavancin. Data collection included demographic information, comorbidities, type of infection, antibiotic usage, IV substance use history and status, presence of hardware, source control interventions, insurance status, and laboratory values, which included complete blood count, comprehensive metabolic panel, and culture data.

The primary outcome was clinical success at 90 days, defined as no need for additional antibiotics (excluding suppression therapy) or infection-related surgical intervention, and no additional positive cultures for the dalbavancin-targeted organism. Secondary outcomes included a safety analysis and an economic impact analysis from the perspective of the health system. The safety analysis evaluated nephrotoxicity, hepatotoxicity, and other antibiotic-related adverse effects. Nephrotoxicity was defined as an increase in serum creatinine by 50% from baseline, and hepatotoxicity was defined as an increase in liver enzymes (AST, ALT, alkaline phosphatase) to three times the upper limit of normal. Other antibiotic-related adverse effects recorded include *C. difficile* infection, vaginal candidiasis, rash, thrush, and infusion-related reactions.

**Figure 1:**
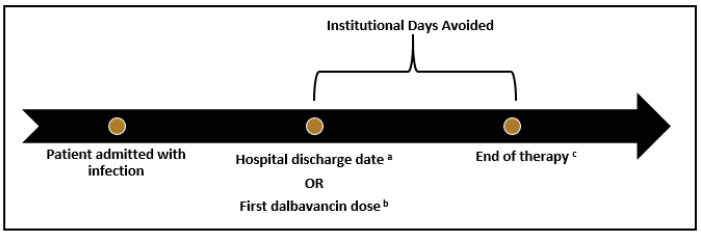
Hospital or SNF days saved calculation; ^a^ after first dalbavancin dose for patients who received dalbavancin inpatient; ^b^ for patients who received first dalbavancin dose as an outpatient; ^c^ 14 days from last dalbavancin dose or end of planned alternative therapy, whichever is shorter.

For the economic analysis, the estimated number of institutional days avoided (IDA) by using dalbavancin instead of standard IV therapy was determined. The start of IDA was either, (1) hospital discharge date for those who received their first dalbavancin dose as an inpatient, or (2) the date of the first dalbavancin dose for those who received their first dose as an outpatient. The end of IDA was determined by adding 14 days to the date of the last dalbavancin dose or it was considered to be the date recorded in the EHR for the end of the planned alternative therapy, whichever was sooner (Figure 1). Cost savings associated with IDA were calculated based on two potential institutional settings, either inpatient hospital location or skilled nursing facility (SNF). The number of IDA was multiplied by the average basic room cost per day for an internal medicine unit at WFBH (USD 472) or the average cost per day at a SNF in Winston-Salem, NC (USD 262) [7]. The cost of dalbavancin was subtracted from savings associated with IDA to determine net financial impact. Based on the mix of patients who were eligible for 340b pricing, a cost of USD 2360 per 1500 mg dose was determined for dalbavancin, and this cost was multiplied by the number of dalbavancin 1500 mg doses per patient before subtracting from the savings associated with IDA. The total cost for the additional hospital or SNF stay was then divided by the total number of patients to determine the estimated cost difference in each treatment location per patient. Due to the inability to anticipate the specific regimens that would have been given instead of dalbavancin, the cost of alternative antibiotics was not incorporated into the analysis, nor were other possible costs associated with a potential hospital stay. Additionally, possible insurance reimbursement was not considered.

Results were analyzed using descriptive statistics. Chi square was utilized for categorical data and Student’s *t*-test or ANOVA was used for continuous variables.

## 3. Results

Forty-eight patients met study criteria and were included in the analysis. Baseline characteristics are summarized in Table 1. The majority of patients were male (56%), the median age was 49 years (range 19 to 93 years), 44% were actively using IV substances, and 15% were self-pay. Dalbavancin was prescribed for osteomyelitis in 26 patients (54%), endocarditis in 11 patients (23%), bacteremia in 7 patients (15%), and prosthetic joint infection in 4 patients (8%). Forty patients (83%) had positive cultures associated with their infections (Table 2). The predominant organism was *Staphylococcus aureus* (60%), and most of these isolates were methicillin-resistant.

The majority of patients (70%) received vancomycin as their initial antibiotic therapy prior to initiating dalbavancin. Other initial antimicrobials included daptomycin, linezolid, clindamycin, doxycycline, cephalexin, rifampin, and sulfamethoxazole/trimethoprim. Most patients (98%) received 1500 mg doses of dalbavancin. In total, 19 patients (44%) received 1 dose, 25 patients (52%) received 2 doses, 3 patients (6%) received 3 doses, and 1 (2%) received 4 doses. While most received all planned doses of dalbavancin, 5 patients (15%) did not complete their entire intended course of therapy. The reason for incomplete therapy was not documented in the EHR. Twelve patients (27%) received concomitant oral antimicrobials (Table 1). The most common oral antimicrobial therapies were fluoroquinolones with 6 patients (13%).

**Figure 2:**
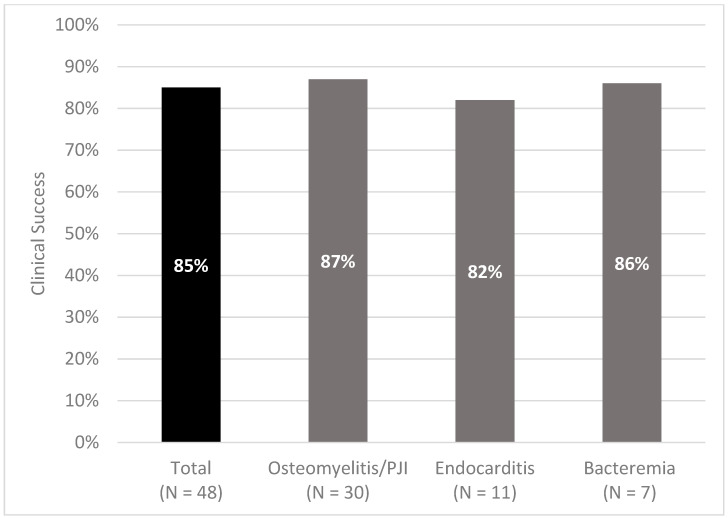
Clinical Success at 90 Days by Indication.

Forty-one patients (85%) achieved the primary outcome of clinical success at 90 days (Figure 2). When broken down by infection type, 22 of 26 patients (87%) with osteomyelitis, 9 of 11 patients (82%) with endocarditis, and 6 of 7 patients (86%) with bacteremia experienced clinical success at 90 days. There was no significant difference in the median length of IV therapy prior to dalbavancin for those who did and did not experience treatment success, 14 versus 11 days, respectively (*p* > 0.5). One patient with bacteremia died prior to the end of the follow-up period from a presumed incurable MRSA infection and cancer. No nephrotoxicity, hepatotoxicity, development of antibiotic resistance, or other antibiotic-related adverse effects were documented.

Collectively, the 48 patients who received dalbavancin accounted for a total of 1056 IDA, or an average of 22 IDA per patient. The net financial impact (savings of IDA minus cost of dalbavancin) was estimated to be USD 1683 and USD 5313 per patient depending on SNF or inpatient hospital location. The average cost of dalbavancin per patients was USD 4080.

## 4. Discussion

These results suggest that dalbavancin therapy has a reasonable success rate and minimal adverse effects when used to treat various off-label, complicated Gram-positive infections. Additionally, dalbavancin therapy may reduce costs when compared to standard regimens by decreasing hospital and/or SNF length of stay.

Osteomyelitis, endocarditis, prosthetic joint infections, and bacteremia can require up to 8 weeks of antimicrobial therapy to fully eradicate the infection. This poses a challenge for patients who are unable or unwilling to receive therapy with long-term IV antibiotics, take oral therapies, or have infections in which oral therapy would be inappropriate. Based on the reasonable success rate of 85%, dalbavancin therapy may be an appropriate antibiotic option for these challenging situations [8].

Previous literature suggest that success rates with dalbavancin for off-label indications are favorable [9]. Most European case series and reports evaluating the efficacy and economic impact of dalbavancin included patients with both on-label and off-label infections. In a retrospective study of 69 patients receiving dalbavancin for Gram-positive infections, Bouza et al. found an 87% success rate with dalbavancin in patients with prosthetic joint infections, bacteremia, osteomyelitis, and skin and soft-tissue infections [10]. Additionally, it was estimated that the use of dalbavancin over standard therapy led to 1160 hospital days saved and an overall cost reduction of EUR 3064 per patient [10]. Similarly, a multicenter, retrospective trial in Austria evaluating dalbavancin for osteomyelitis, prosthetic joint infection, endocarditis, and skin and soft-tissue infections found a clinical success rate of 89% with minimal adverse effects [11].Finally, a German study evaluating potential cost savings of single-dose dalbavancin estimated that dalbavancin saved 6.45 hospital days and EUR 2865 in patients with MRSA skin and soft-tissue infection and 10.6 hospital days and EUR 3909 in patients with MRSA bone and joint infections [12].

In a 2019 retrospective study completed at the University of Colorado Health, it was found that 15% of patients treated with dalbavancin or oritavancin for on- and off-label indications experienced clinical failure. In this study, it was predicted that the use of dalbavancin or oritavancin would lead to approximately 9 hospital days and USD 17,204 saved per patient. Finally, a prospective, randomized trial by Rappo et al. looked specifically at dalbavancin for osteomyelitis. This trial showed a clinical response rate of 97% at day 42 [6]. Unlike the previously available literature, our study focused only on the off-label use of dalbavancin, but demonstrated similar rates of clinical success. Previous data combined with the findings of this study suggest that dalbavancin is a reasonable treatment option for these infections.

The model for calculating potential cost savings in this study was very conservative when compared to other studies evaluating cost saving with dalbavancin therapy. Unlike some other studies, we included cost figures, and not patient charges in our model. We only included the actual base cost of hospitalization for our facility (USD 427/day) in our analysis, realizing that it would likely underestimate true cost avoidance. In the previously mentioned study completed at The University of Colorado Health, their estimated cost of hospitalization per day of USD 2090 was based on the average expenses per inpatient day in the United States reported by the Kaiser Family Foundation for the year 2012 [4,5,7]. If we used a similar approach and incorporated the current average expense figure from the Kaiser Family Foundation (USD 2607/day) into our analysis, the calculated cost avoidance from reduced hospital length of stay would be approximately USD 53,000 per patient. Since it is unreasonable to speculate where each individual would have received alternative therapy, our analysis estimated cost savings for both SNF and hospital stay for all patients. Finally, possible insurance reimbursement was not considered. In summary, although it is unrealistic to predict the exact cost savings associated with the use of dalbavancin for off-label indications, its use is likely to result in cost savings even higher than our conservative model predicted. As hospitals continue to face pressure to reduce expenses, the use of dalbavancin to facilitate early discharge may safely reduce overall expenditures for select patients.

This study has limitations worth noting. Due to the retrospective nature of this study, data collection was dependent upon accurate documentation in the EMR, and we were only able to capture data within our EMR’s network. Additionally, this study was conducted within a single health system whose stewardship program placed restrictions on dalbavancin to ensure appropriate use [13]. Outcomes may not be reproducible at institutions that do not have the same level of stewardship oversight. Finally, the cost impact analysis is only an estimate since it is challenging to predict exactly what would have happened if dalbavancin was not used in these patients.

## 5. Conclusions

In conclusion, our study adds to the existing literature demonstrating reasonable success rates and minimal adverse effects with the use of dalbavancin in the treatment of various off-label, Gram-positive infections. Additionally, dalbavancin has the potential to reduce cost when compared to standard therapy. In the future, randomized, controlled trials would be beneficial to delineate the role of dalbavancin in the treatment of various off-label infections and its impact on resource utilization.

## Figures and Tables

**Table 1:** Patient Characteristics (N = 48).

Baseline Characteristic	
Male, n (%)	27 (56)
Age (years), median [range]	49 [19–93]
IV substance user, n (%)	21 (44)
Immunocompromised, n (%)	6 (13)
Diabetes, n (%)	21 (44)
Treating a recurring infection, n (%)	11 (23)
Self-pay, n (%)	7 (15)
BMI (kg/m^2^), median [range]	26.7 [16.7–57]
**Initial Antibiotics**	**n (%)**
Vancomycin	35 (73)
Daptomycin	4 (8)
Linezolid	2 (4)
Other	7 (15)
**Concomitant Oral Antimicrobials with Dalbavancin**	**n (%)**
Rifampin plus fluoroquinolone	5 (10)
Doxycycline	3 (6)
Sulfamethoxazole/Trimethoprim	2 (4)
Amoxicillin plus ciprofloxacin	1 (2)
Cephalexin	1 (2)
Ciprofloxacin	1 (2)
Rifampin	1 (2)
None	34 (71)

**Table 2:** Infection Characteristics (N = 48).

Type of Infection	n (%)
Osteomyelitis	26 (54)
Endocarditis	11 (23)
Bacteremia	7 (15)
Prosthetic Joint Infection	4 (8)
**Pathogen**	**n (%)**
Methicillin-Resistant *Staphylococcus aureus* (MRSA)	20 (42)
Methicillin-Susceptible *Staphylococcus aureus* (MSSA)	9 (19)
No Positive Culture	8 (17)
Polymicrobial	5 (10)
Coagulase-Negative *Staphylococcus*	3 (6)
*Corynebacterium*	2 (4)
*Enterococcus faecalis*	1 (2)

## Data Availability

Not applicable.
